# Future Prospects of Imatinib in Advanced Pulmonary Hypertension Management

**DOI:** 10.1002/iub.70098

**Published:** 2026-03-26

**Authors:** Federica Davì, Stella Mangione, Antonella Iaconis, Tiziana Genovese, Nicla Tranchida, Salvatore Cuzzocrea

**Affiliations:** ^1^ Department of Chemical, Biological, Pharmaceutical and Environmental Sciences University of Messina Messina Italy; ^2^ Azienda Ospedaliera Universitaria Policlinico AOU G. Martino Messina Italy

**Keywords:** imatinib, pharmacological treatment, pulmonary arterial hypertension (PAH), pulmonary vascular resistance, survival, tyrosine kinase inhibitors, vascular remodeling

## Abstract

Pulmonary arterial hypertension (PAH) is a severe, progressive disease characterized by elevated pulmonary arterial pressure and increased vascular resistance. This hemodynamic strain forces the right ventricle to pump against a high‐pressure system, ultimately leading to right‐sided heart failure and death. The pathogenesis of PAH involves a complex interplay of vasoconstriction, chronic inflammation, and pathological remodeling of the pulmonary vessel walls—specifically hypertrophy of the smooth muscle and intimal layers—driven by molecular imbalances and genetic predispositions. Current FDA‐approved therapies primarily manage symptoms through vasodilation but fail to directly target the underlying vascular remodeling. Imatinib, a tyrosine kinase inhibitor originally developed for oncological indications, has emerged as a potential disease‐modifying agent for PAH. By inhibiting platelet‐derived growth factor receptors (PDGFR), imatinib targets the aberrant proliferation of smooth muscle cells, offering a mechanism to potentially reverse or arrest vascular remodeling. Clinical trials, including the IMPRES study, have demonstrated encouraging hemodynamic improvements in patients with severe PAH refractory to standard therapies. However, systemic safety concerns and dose‐dependent adverse reactions have limited its clinical approval. This review examines the pharmacological rationale for imatinib, its impact on vascular structure, and the safety signals observed in long‐term studies. Furthermore, it discusses emerging strategies, such as inhaled formulations and pharmacogenetic approaches (e.g., the PIPAH study), aimed at enhancing the efficacy‐to‐safety ratio of kinase inhibitors to improve long‐term outcomes for patients with PAH.

## 1 Introduction

Pulmonary hypertension (PH) is a clinical condition affecting approximately 1% of the world's population [1]. It is characterized by abnormally high pulmonary artery pressure values [2], with current guidelines setting 20 mmHg as the upper limit of normal for mean pulmonary artery pressure (mPAP) [1, 3, 4]. PH is not merely a hemodynamic finding; over time, persistently high pressures and increased resistance in the pulmonary vessels strain the right side of the heart, potentially leading to right heart failure [5].

The pathogenesis of PH involves multiple interacting mechanisms, including vasoconstriction, endothelial dysfunction, inflammation, and, most critically, vascular remodeling. Together, these processes slowly compromise blood flow through the lungs and place an increasing workload on the right ventricle. While vascular remodeling may initially be the body's way of coping with high pressures, it eventually becomes a maladaptive process that drives disease progression [6, 7].

**FIGURE 1:**
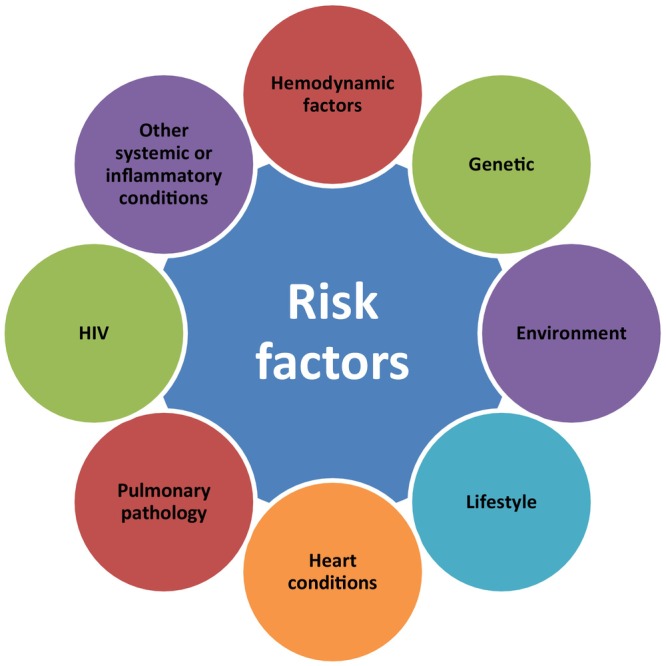
Risk factors of PH.

A variety of factors contribute to the development of PH, guiding its clinical classification into five distinct groups (Figure 1 and Table 1). Hemodynamic factors arising from heart conditions, such as left‐sided valve disease or chronic heart failure, lead to post‐capillary PH (Group 2) [9].

**TABLE 1:** Classification by WSPH [8].

Category 1	PAH
Category 2	PH associated with left heart disease
Category 3	PH associated with lung disease and/or hypoxia
Category 4	Chronic thromboembolic pulmonary hypertension
Category 5	PH with unclear or multifactorial mechanisms

Pulmonary pathologies, including COPD, pulmonary fibrosis, and obstructive sleep apnea, are major contributors to PH associated with lung disease (Group 3) [9]. Additionally, blood clots or chronic thromboembolism increase the risk of chronic thromboembolic pulmonary hypertension (Group 4). Finally, Group 5 encompasses PH with unclear or multifactorial mechanisms. Other systemic conditions, such as HIV infection, autoimmune diseases, or liver disease, can also drive the pathology [10, 11, 12].

While Groups 2 and 3 account for the majority of cases worldwide (90%–95%) [1, 3, 13], Pulmonary Arterial Hypertension (PAH, Group 1) represents a distinct clinical and pathological entity. PAH is primarily driven by a progressive vasculopathy of the small pulmonary arteries, often linked to genetic mutations in genes such as *BMPR2*, *ALK1*, and *KCNK3* [14].

PH often develops quietly, with exertional dyspnea being the most telling early symptom [15]. As the disease progresses, fatigue, syncope, and signs of systemic venous congestion—such as edema or ascites—become evident [1, 13]. Because these symptoms overlap with common conditions like asthma or COPD, PAH is frequently diagnosed at an advanced stage [1, 3]. Early recognition and accurate classification are therefore crucial for survival. Hemodynamic classification remains the gold standard [1, 3]:

1. Pre‐capillary PH (PAH profile): mPAP > 20 mmHg, pulmonary vascular resistance (PVR) > 2 Wood units, and pulmonary artery wedge pressure (PAWP) ≤ 15 mmHg [1, 3, 4, 16].
2. Post‐capillary PH: mPAP >20 mmHg, PAWP > 15 mmHg, typically secondary to left heart disease.
3. Exercise‐induced PH: normal resting mPAP, with abnormal increases during physical activity (mPAP/CO slope > 3 mmHg/L/min).

Accurate differentiation remains challenging, especially as the majority of PAH patients are now over 60 years of age and present with multiple comorbidities [17]. A PAWP value ≤ 15 mmHg is essential to define Group 1; however, subclinical left heart disease cannot always be excluded by resting measurements alone [18, 19, 20]. Consequently, a comprehensive diagnostic workup—including pulmonary function testing and high‐resolution CT—is necessary to distinguish PAH from PH associated with lung or cardiac disorders.

Despite the diagnostic framework provided by the WSPH and the 2022 ESC/ERS guidelines, the management of PAH remains difficult because current therapies primarily target vasodilation rather than structural remodeling. This review aims to critically analyze the dual face of imatinib in PAH: its promising antiproliferative effects—targeting the underlying vascular remodeling—versus its problematic safety profile, exploring future avenues for its optimal use.

## 2 Genetics

Genetic predisposition plays a central role in the pathogenesis of PAH. Certain genetic mutations, such as those involving the *BMPR2* gene, have been shown to be associated with heritable pulmonary arterial hypertension (HPAH), supporting the existence of a clear correlation between genetic background and clinical‐pathological manifestations [1]. In 2023, an expert group released the International Consensus Statement on Genetic Counselling and Testing in Pulmonary Arterial Hypertension, endorsed by the European Respiratory Society (ERS) and the Pulmonary Vascular Research Institute (PVRI). This document recommended expanding the list of genes recognized as contributing to the genetic predisposition to PAH [21]. Among these, *TBX4*, *SOX17*, and *KDR* play crucial roles in pulmonary angiogenesis as well as in the development of lung parenchyma, bronchi, and the heart. Variations in these genes give rise to multiple phenotypic manifestations, which may include congenital heart defects and severe abnormalities in lung development [1]. The earliest hypotheses proposing a link between genetic factors and familial forms of PAH date back to the 1950s. However, the pivotal discoveries emerged in the 1990s, when studies identified pathogenic germline mutations in the *BMPR2* gene as the primary cause of heritable PAH [22]. Subsequent genetic investigations provided additional evidence implicating other genes in genetic susceptibility to the disease [22]. More recently, international research efforts have identified twelve genes that are now recognized as being strongly associated with predisposition to PAH: *BMPR2*, *ACVRL1*, *ATP13A3*, *CAV1*, *EIF2AK4*, *ENG*, *GDF2*, *KCNK3*, *KDR*, *SMAD9*, *SOX17*, and *TBX4*. In contrast, current evidence regarding the involvement of *ABCC8*, *GGCX*, and *TET2* remains inconclusive, and further studies are needed to clarify their potential association with PAH [23].

## 3 Pathogenesis

**FIGURE 2:**
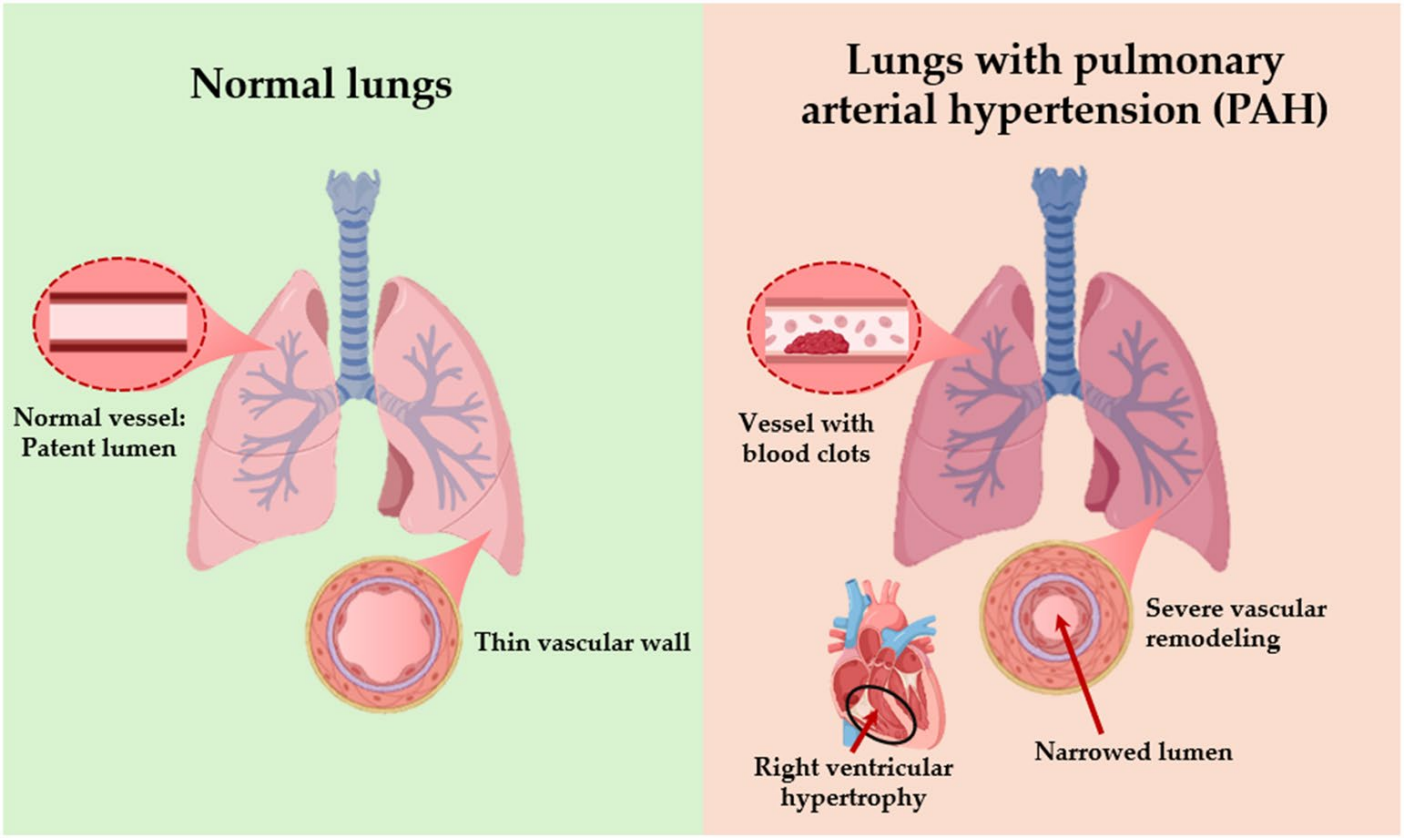
Comparative pathophysiology of normal pulmonary vasculature versus PAH. *Left*: Normal lung with thin‐walled arteries and wide lumen. *Right*: PAH lung demonstrating severe vascular remodeling, including medial hypertrophy, intimal thickening (onion‐skin lesions), and the presence of in situ blood clots (microthrombosis) obstructing the vessel. Note the secondary right ventricular hypertrophy (RVH) resulting from increased pulmonary vascular resistance.

While pulmonary hypertension (PH) generally puts a strain on both the heart and lungs, the specific pathogenic mechanisms are most clearly defined in Pulmonary Arterial Hypertension (PAH). The disease begins when the pressure in the pulmonary arteries rises and the resistance within these vessels increases (Figure 2). Over time, this constant stress forces the right side of the heart to work harder, often leading to right heart failure [16].

The mechanisms behind PAH are complex and interconnected, involving vasoconstriction, structural changes in the vessel walls (vascular remodeling), endothelial dysfunction, inflammation, and clot formation. In addition, chronic inflammation and oxidative stress play a significant role in disease progression: immune cells infiltrate the vessel wall, releasing cytokines that further drive vascular remodeling, while reactive oxygen species (ROS) accumulate, reducing nitric oxide availability and promoting abnormal proliferation of smooth muscle and endothelial cells [24, 25, 26, 27, 28].

At first, these changes are adaptive responses to increased pressure. However, they eventually become pathological, stiffening and narrowing the vessels [6, 7]. The earliest visible changes occur in the middle layer of the pulmonary arteries, where smooth muscle cells thicken and multiply, and previously non‐muscularized arterioles develop muscular walls [6, 29]. Key molecular signals—such as platelet‐derived growth factor (PDGF) and transforming growth factor‐β—push these cells into overdrive encouraging growth and abnormal behavior [25]. The innermost layer of the vessels, the intima, also undergoes significant changes: endothelial cells multiply and fibrotic tissue builds up, sometimes forming concentric “onion‐skin” lesions that narrow the lumen [7, 29]. Even the outer layer, the adventitia, is active, with fibroblast expansion and inflammatory cell infiltration [7]. Oxidative stress and inflammation in the adventitia further amplify these pathological changes, contributing to endothelial dysfunction and promoting the formation of plexiform lesions [26, 30, 31, 32, 33]. In the most severe cases, like idiopathic pulmonary arterial hypertension (IPAH), tangled endothelial growths known as plexiform lesions appear, representing pathological new vessel formation [6, 7]. Vasoconstriction adds to the problem: endothelial cells fail to produce enough nitric oxide (NO) and prostacyclin (PGI_2_), which normally relax vessels, while levels of endothelin‐1 (ET‐1)—a potent constrictor—rise [29]. Together, these changes stiffen the vessels, narrow the lumen, and increase pulmonary artery pressure, creating a chronic overload for the right ventricle [7]. Among PH subtypes, PAH has been studied the most. In PAH, the arteries in the lungs become stiffer and narrower, making it difficult for blood to pass through. This increases pulmonary vascular resistance and forces the right heart to pump harder [27]. Three molecular pathways play a major role: reduced nitric oxide, decreased prostacyclin (PGI₂), and overactive endothelin‐1 signaling [28]. The interplay between oxidative stress, inflammation, and these pathways underscores the multifactorial nature of PAH and highlights potential targets for novel therapeutic strategies [26, 30, 31, 32, 33]. Understanding these pathways has allowed the development of targeted treatments, although the persistent challenge of vascular remodeling suggests that addressing cellular proliferation through drugs like imatinib is a necessary evolution in PAH management.

### 3.1 Nitric Oxide Pathway

Nitric oxide (NO) is a molecule produced by endothelial cells that keeps vessels relaxed and healthy. Using oxygen and cofactors, the enzyme endothelial nitric oxide synthase (eNOS) converts L‐arginine into NO, which diffuses into the smooth muscle of pulmonary vessels. There, it triggers a chain reaction that relaxes the muscle, widens the vessels, and prevents cell overgrowth, clot formation, and inflammation.

In PAH, NO levels drop, causing blood vessels to tighten, smooth muscle cells to proliferate, and the lungs' microvasculature to become inflamed and prone to clotting. Interestingly, this can happen both when eNOS is underactive or overactive, due to oxidative stress and cofactors like tetrahydrobiopterin (BH_4_) being depleted [34, 35]. Treatments targeting this pathway include:

- PDE‐5 inhibitors, which prevent NO breakdown and enhance its effects.
- sGC stimulators, which increase cGMP even when NO is low.

### 3.2 Prostacyclin–Thromboxane A₂ Pathway

Prostacyclin is another molecule that relaxes vessels and prevents platelets from clumping. It is produced by endothelial cells and binds to receptors on smooth muscle, causing the vessel to widen. It also has anti‐inflammatory and antiproliferative effects. In PAH, this balance shifts toward thromboxane A_2_ (TXA_2_), which constricts vessels, promotes platelet aggregation, and encourages cell growth. Animal studies show that mice lacking prostacyclin receptors develop severe PAH when exposed to chronic low oxygen [36]. In patients, reduced prostacyclin production and receptor expression are observed. Treatments include prostacyclin analogues and receptor agonists.

### 3.3 Endothelin‐1 Pathway

Endothelin‐1 (ET‐1) is a potent constrictor produced by endothelial cells. It acts on two receptors:

ET_A_, on smooth muscle cells, causes vasoconstriction, cell growth, and fibrosis; ET_B_, found on smooth muscle and endothelial cells, where on smooth muscle it constricts, while on endothelium it stimulates NO and prostacyclin to dilate vessels. In PAH, ET‐1 levels are high, ET_A_ and ET_B_ receptors on smooth muscle are upregulated, and protective endothelial ET_B_ expression drops [37]. Drugs called endothelin receptor antagonists (ERAs) block these effects:

- Selective ET_A_ antagonists, which target the constrictive receptor.
- Dual ET_A_/ET_B_ antagonists, which block both receptors, restore balance between constriction and dilation.

Identifying these complex pathological changes early is vital, yet the diagnostic journey for PAH remains a significant clinical challenge.

## 4 Diagnosis

The gold standard for the diagnosis of pulmonary hypertension is right heart catheterization (RHC), which allows direct measurement of mean pulmonary arterial pressure and pulmonary vascular resistance [28]. To minimize diagnostic delays and ensure accuracy, the 2022 ESC/ERS guidelines recommend a standardized five‐step diagnostic algorithm [1]:

### 4.1 First Step: Clinical Assessment

Evaluation begins when symptoms such as dyspnea, fatigue, or signs of right heart failure are present. Associated conditions, including connective tissue disease, HIV infection, or congenital heart disease, must be considered. A detailed history of current or previous use of anorexigenic or stimulant drugs is essential, as these are recognized risk factors for pulmonary arterial hypertension (PAH) [1]. Physical examination findings, such as an accentuated second heart sound (P_2_) or a tricuspid regurgitation murmur, further raise clinical suspicion.

### 4.2 Second Step: Basic Diagnostic Tests

Initial non‐invasive tests include electrocardiography (ECG), chest radiography, BNP/NT‐proBNP assay, and routine blood analyses [2]. ECG findings such as right axis deviation, right ventricular hypertrophy, or right atrial enlargement are suggestive of PH. Conversely, normal ECG and BNP results generally indicate a low likelihood of the disease [1, 34].

### 4.3 Third Step: Cardiac and Pulmonary Imaging

Echocardiography is the principal screening tool for PH. The measurement of tricuspid regurgitant velocity (TRV) provides a probabilistic estimate of the disease:

TRV > 3.4 m/s → high probability.

TRV 2.9–3.4 m/s → intermediate probability.

TRV < 2.8 m/s → low probability [1, 34].

High‐resolution computed tomography (CT) is preferred over chest radiography for detecting underlying lung parenchymal diseases, helping to differentiate between Group 1 (PAH) and Group 3 PH [38]. A low diffusing capacity for carbon monoxide (DLCO < 40%) or centrilobular ground‐glass opacities may suggest specific pathologies like pulmonary veno‐occlusive disease (PVOD) [39, 40]. These findings reflect impaired alveolar‐capillary gas exchange and possible underlying pulmonary pathology. In recent years, artificial intelligence–based imaging technologies have significantly enhanced diagnostic accuracy and efficiency [41, 42].

### 4.4 Fourth Step: V′/Q′ Scintigraphy and Laboratory Evaluation

Ventilation/perfusion (V′/Q′) scintigraphy is the key diagnostic test for excluding chronic thromboembolic pulmonary hypertension (CTEPH, Group 4). Normal ventilation with segmental perfusion defects is highly suggestive of this condition [43]. Further laboratory testing is required to screen for thyroid dysfunction, autoimmune markers, HIV, and viral hepatitis. Assessment for methamphetamine‐associated PAH via urinary toxicology screening is also recommended in specific clinical contexts [44].

### 4.5 Fifth Step: Referral and Invasive Testing

Patients with an intermediate or high probability of PH should be referred to specialized centers for confirmatory RHC. Beyond confirming the diagnosis, additional investigations such as acute vasoreactivity testing—essential for identifying candidates for calcium channel blocker therapy in PAH—or fluid challenges may be performed based on the patient's hemodynamic profile [1].

## 5 Approved Therapies for the Treatment of Pulmonary Arterial Hypertension (PAH)

Several pharmacological classes have been approved by the U.S. Food and Drug Administration (FDA) specifically for the treatment of Pulmonary Arterial Hypertension (PAH) [45, 46]. These agents are categorized into four primary groups based on their targeted molecular pathways [45]:

1. *Prostacyclin analogues* [36, 47]: These agents, including epoprostenol, iloprost, and treprostinil, compensate for the endogenous deficiency of prostacyclin observed in PAH. By promoting pulmonary vasodilation and inhibiting platelet aggregation, they reduce right ventricular afterload and improve exercise capacity. Administration routes vary from continuous intravenous or subcutaneous infusion to inhalation and oral tablets. Common adverse effects include headache, flushing, nausea, and hypotension.
2. *Prostacyclin receptor agonists* [36, 47]: Selexipag is the primary non‐prostanoid IP receptor agonist. Unlike direct analogues, it selectively targets the prostacyclin receptor to induce vasodilation and anti‐proliferative effects. Its oral availability offers a less invasive alternative for patients, though it requires careful dose titration due to potential vasodilator‐related side effects.
3. *Phosphodiesterase type 5 (PDE‐5) inhibitors* [47, 48]: Sildenafil and tadalafil increase the bioavailability of cyclic GMP by inhibiting its degradation by the PDE‐5 enzyme. This enhances the nitric oxide‐mediated vasodilator signal, leading to improved pulmonary hemodynamics and exercise tolerance. While generally well‐tolerated, they may cause nasal congestion, headaches, and systemic hypotension.
4. *Endothelin receptor antagonists* [47, 49]: This class, including bosentan, macitentan, and ambrisentan, blocks the overactive endothelin‐1 pathway. By antagonizing ET_A_ and/or ET_B_ receptors, these drugs mitigate vasoconstriction and vascular wall thickening. Clinical monitoring is required due to risks of fluid retention, potential hepatotoxicity (primarily with bosentan), and teratogenicity, making them contraindicated during pregnancy.

### 5.1 Drug Associated With PAH

Several pharmacological agents are recognized for their potential to induce or contribute to PAH. This list is periodically updated by the World Symposium on Pulmonary Hypertension (WSPH) and the European Respiratory Society/European Society of Cardiology (ERS/ESC) guidelines. The current classification simplifies the strength of evidence into two categories [2]:

- Definite Association: Established through substantial evidence, such as case–control studies, multi‐centre case series, or documented drug‐related epidemics (e.g., aminorex or methamphetamines).
- Possible Association: Assigned to drugs with sharing pharmacological mechanisms with known PAH‐inducers or where evidence is limited to isolated clinical cases.

The complexity of establishing these causal links arises from confounding factors such as the latency period between exposure and disease onset, the variable subset of subjects affected, and the degree of PAH reversibility upon drug discontinuation. Table 2 provides a comprehensive synthesis of these associations, incorporating the classification of drugs and toxins associated with PAH (adapted from the 6th WSPH) and aligned with the latest ERS/ESC guidelines and recent clinical reviews [8, 50, 51].

**TABLE 2:** Drugs and toxins associated with the development of pulmonary arterial hypertension (PAH) [8, 50, 51].

Certain association	Possible association
Aminorex	Alkylating agents
Benfluorex	Amphetamines
Carfilzomib	Bevacizumab
Desatinib	Bortezomib
Dexfenfluramine	Bosutinib
Fenfluramine	Cocaine
Methamphetamines	Diazoxide
Mitomycin C	Direct‐acting antiviral agents against hepatitis C virus (sofosbuvir)
Toxic rapeseed oil	Indigo naturalis (Chinese herb Qing‐Dai)
	Interferon‐α and ‐β
	Leflunomide
	l‐tryptophan
	Phenylpropanolamine
	Ponatinib
	Solvents (trichloroethylene) St John's wort

### 5.2 Emerging Therapies

Recent research has pivoted from pure vasodilators toward agents targeting vascular remodeling in PAH, often repurposing drugs from other therapeutic areas. Among these, imatinib, originally a tyrosine kinase inhibitor (TKI) for chronic myeloid leukemia, has demonstrated potential in addressing the proliferative signaling that drives abnormal lung vessel remodeling.

By inhibiting platelet‐derived growth factor (PDGF) signaling, imatinib targets the hyper‐proliferation of smooth muscle cells within the pulmonary vasculature. While it is not yet approved as a first‐line treatment for PAH due to its safety profile, it remains a critical proof‐of‐concept for the use of TKIs in managing refractory disease. Table 3 lists eight novel drugs currently under investigation for their efficacy in treating PAH [36].

**TABLE 3:** Summary of emerging and investigated pharmacological agents for the treatment of pulmonary arterial hypertension (PAH).

Drug name	Mechanism of action and clinical findings
ANASTROZOLE	‐Inhibits aromatases [48] ‐Reduces estradiol level [52] ‐Improves 6‐MWD values [52] ‐Has no effect on TAPSE and does not affect quality of life [52] ‐Reduces pulmonary pressure [52, 53] ‐Reduces right ventricular hypertrophy [52, 54] ‐It appears to be a safe drug overall, however, its study still needs further investigation [52]
IMATINIB	‐oral kinase inhibitor drug ‐Improves haemodynamics and vascular resistance in patients with refractory pulmonary hypertension [53, 55] ‐Serious adverse reactions and several deaths have occurred, leading to dropout from the study ‐It is therefore not suitable for the treatment of PAH [55].
RACECADOTRIL	‐Inhibitor of neprisilin ‐Increases the bioavailability of c‐GMP ‐Increases the bioavailability of vasodilator peptides ‐In patients already receiving PDE‐5i therapy, reduced PVR and increased c‐GMP and ANP [56] ‐Showed no major adverse reactions [56] ‐Also used in cases of diarrhea [57] [58] ‐Further studies are needed to firmly establish its use in the treatment of PAH [56]
RALINEPAG	‐Prostacyclin receptor agonist [58] ‐Has a half‐life of 24 h [58] ‐Reduces PVR in patients already being treated for PAH with basic therapy [59] ‐Slight increase in 6‐MWD ‐Good pharmacological safety ‐Study still needs to be integrated and further Investigated [59]
RIOCIGUAT	‐Activates soluble guanylate cyclase ‐Increases c‐GMP resulting in vasodilation [60] ‐Approved drug for the treatment of pulmonary hypertension [61] ‐Has produced improvements in 6‐MWD and PVR [62] ‐Several studies have confirmed its efficacy for the treatment of PAH [63]
SOTATERCEPT	‐Is an experimental fusion protein ‐improves ISR and 6‐MWD in subjects already receiving basic therapy for PAH [64] ‐This drug was also studied in the past for the treatment of other diseases such as β‐thalassaemia and various anaemias [65, 66, 67, 68, 69, 70, 71] ‐Further studies on it are needed to confirm it as a drug for PAH [64]
TACROLIMUS	‐Starts the BMPR2 signaling pathway resulting in reversal of vasculopathy in animals with pulmonary hypertension [72] ‐The clinical parameters of several patients with advanced PAH [73] ‐In other studies with patients with stabilized PAH, low‐dose tacrolimus did not cause problems by increasing activation of the BMPR2 pathway in PBMCs, however, no significant benefit was found [74] ‐Further studies are needed [74]
UDENAFIL	‐PDE‐5 inhibitor ‐Has a long half‐life ‐Improves vasodilation 12 h after intake [75] ‐Fifty milligrams administered was sufficient to significantly reduce pulmonary vascular resistance, compared to 100 [76] ‐After 16 weeks, the 50 mg dose further improved the 6‐min distance travelled especially in subjects who were already being treated with ERA [77] ‐Appears to be a fairly efficient drug but also needs more study and investigation [77]

While these emerging therapies aim to address various pathogenic pathways, imatinib stands out as the most extensively studied agent with a distinct disease‐modifying potential. The therapeutic gap between existing vasodilators and the need for structural arrest has driven a deep clinical interest in imatinib, which we will analyze in detail in the following sections to explore its future prospects.

## 6 Imatinib: A Targeted Strategy for Vascular Remodeling

**FIGURE 3:**
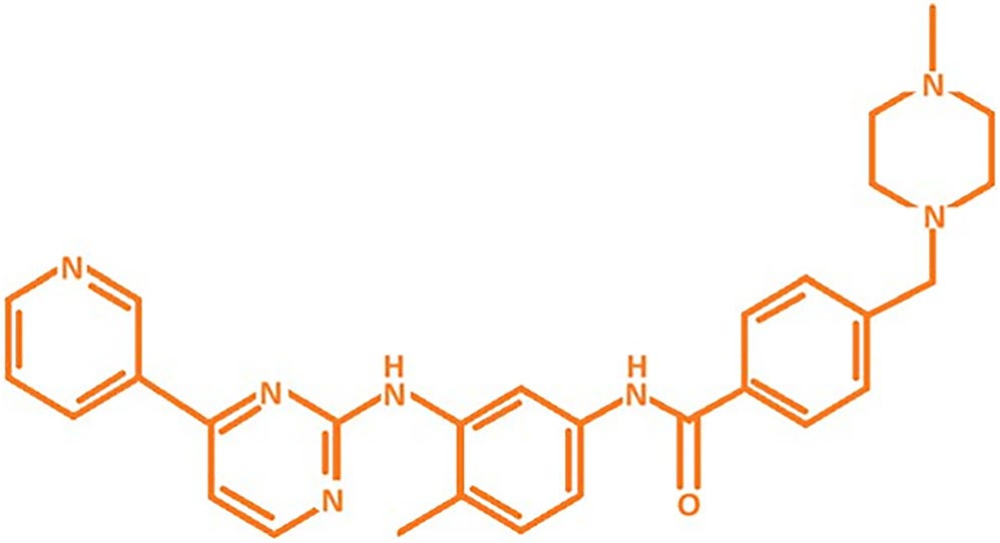
Molecular structure of imatinib.

Imatinib (Figure 3) is a potent tyrosine kinase inhibitor (TKI) that targets the Bcr‐Abl protein, the KIT receptor, and, most importantly for pulmonary vascular disease, the platelet‐derived growth factor receptors (PDGFRα and PDGFRβ) [78, 79]. While originally designed to block malignant cell proliferation by selectively inhibiting tyrosine kinases, its ability to arrest the abnormal growth of smooth muscle cells within the vascular wall makes it a compelling candidate for reverse remodeling in PAH.

Activation of the PDGF pathway has been demonstrated both in patients and in pulmonary hypertension (PH) experimental models, where four different isoforms of PDGF control vascular cell migration, proliferation, and survival [80]. These PDGFs elicit multiple intracellular signaling pathways specifically within pulmonary smooth muscle cells, establishing the PDGFR as a primary target for disease‐modifying intervention [80]. In contrast to traditional vasodilators, targeting this pathway aims to fundamentally alter the pulmonary vessel architecture rather than just modulating vascular tone.

Translating these molecular effects into clinical success requires a detailed understanding of the drug's behavior within the body, as its pharmacokinetic profile directly influences both efficacy and systemic safety in PAH patients.

From a pharmacokinetic perspective, imatinib exhibits rapid oral bioavailability that is not significantly affected by food intake. It has a half‐life of approximately 18 h and is highly protein‐bound (95%), primarily to albumin and α1‐acid glycoprotein. Metabolism is predominantly hepatic, mediated by the CYP3A4 and CYP3A5 enzymes. Notably, imatinib also acts as a competitive inhibitor of these enzymes, creating a significant potential for drug–drug interactions with other PAH therapies metabolized via the same pathway [81]. Elimination is primarily biliary, with a feces‐to‐urine excretion ratio of approximately 5:1.

Historically, these inhibitory mechanisms have been utilized to treat chronic myeloid leukemia (CML) and gastrointestinal stromal tumors (GIST). However, the therapeutic focus in this review is how these same pathways—specifically PDGFR inhibition—can address the structural vascular changes characteristic of advanced PAH.

This shift from oncology to pulmonary vascular science necessitates a rigorous evaluation of how imatinib interacts with the unique physiological environment of the PH patient.

### 6.1 Pharmacokinetic Challenges in the PAH Patient

Imatinib exhibits high solubility in acidic environments, facilitating rapid oral absorption. Clinical guidelines recommend administration with water and food to mitigate gastrointestinal side effects, as food intake does not significantly impact its overall bioavailability [81]. Peak plasma concentrations (*C*_max_) of approximately 1.9 μg/mL are achieved within 2–4 h post‐dose, with steady‐state levels reached after repeated daily administration of 400 mg.

The drug's distribution and metabolism are characterized by several key factors [81]:

- Protein Binding: As previously noted, imatinib is 95% bound to plasma proteins. While α 1‐acid glycoprotein levels can vary in different disease states, this does not substantially alter the drug's pharmacokinetic profile.
- Hepatic Metabolism: Metabolism occurs extensively in the liver via the cytochrome P450 system, primarily CYP3A4 and CYP3A5. The main active metabolite, an N‐demethylated piperazine derivative, accounts for 10%–15% of the total area under the curve (AUC) and maintains a pharmacological potency similar to the parent compound. Crucially, in PAH patients, hepatic metabolism of imatinib may be further complicated by right heart failure‐induced liver congestion, which can impair cytochrome P450 activity and lead to unpredictable drug accumulation [50].
- Elimination: The terminal half‐life of imatinib is approximately 19 h, while its primary metabolite possesses a longer half‐life of about 40 h. Biliary excretion is the dominant pathway, with roughly 68% of the dose eliminated in feces as metabolites and 20% as unchanged drug; renal clearance remains minimal.

#### 6.1.1 Drug–Drug Interactions

The metabolic reliance on the CYP3A4 pathway renders imatinib highly susceptible to interactions, a factor of paramount importance in the PAH population. CYP3A4 inhibitors (e.g., ketoconazole, erythromycin) can elevate imatinib plasma levels, increasing toxicity risks, while CYP3A4 inducers (e.g., rifampin, St. John's wort) may lead to subtherapeutic exposure. Furthermore, as an inhibitor of CYP3A4 itself, imatinib can increase the systemic concentration of co‐administered agents like simvastatin or cyclosporine. In clinical practice, PAH management often requires the co‐administration of multiple drugs to target several pathogenic mechanisms simultaneously [82]. While such combination treatments aim to exploit synergism and allow for dose reductions, they significantly increase the clinical burden of potential drug–drug interactions [82]. Such interactions can compromise treatment efficacy or exacerbate side effects by altering drug transport, absorption, and excretion. Therefore, predicting DDIs is essential in PAH patients to ensure that the addition of imatinib to standard therapies does not negatively influence disease progression or health care costs [82].

In terms of patient demographics, age and hepatic impairment do not appear to significantly necessitate dose adjustments. However, in patients with renal dysfunction, a notable reduction in drug clearance has been observed, requiring closer clinical monitoring [81].

### 6.2 Pharmacogenetic Determinants of Toxicity and Response

Beyond general metabolic pathways, the inter‐individual variability observed in clinical trials has shifted focus toward specific genetic determinants. Genetic polymorphisms in cytochrome P450 (CYP) enzymes, primarily involved in imatinib metabolism, could theoretically alter the drug's pharmacokinetics. However, clinical studies have demonstrated that polymorphisms in CYP3A4, CYP3A5, and other isoforms (such as CYP2C9 and CYP2C19) do not cause significant alterations in imatinib exposure. This lack of impact is likely due to high interindividual variability in enzyme expression and the relatively low prevalence of these specific gene variants [83]. Consequently, the research spotlight has shifted from hepatic enzymes to drug transport mechanisms. A more relevant area of current investigation involves the ATP‐binding cassette (ABC) transporters, specifically ABCB1 (P‐glycoprotein) and ABCG2 (Breast Cancer Resistance Protein). These transporters actively mediate biliary excretion, which—as noted in previous sections—is the principal elimination pathway for imatinib. Understanding how genetic variations in these transporters affect drug concentration is crucial, as they could influence both the efficacy and the systemic toxicity of the treatment in PAH patients [84]. Importantly, genetic variations in ABCB1 and ABCG2 are not only relevant for systemic clearance but may also dictate the local concentration of imatinib within the pulmonary endothelium. This localized accumulation potentially contributes to the vascular fragility and severe bleeding risks—such as subdural hematomas—observed in advanced PAH cohorts during clinical trials [85].

### 6.3 Mechanisms of Reduced Sensitivity and Resistance in PAH

Understanding the long‐term impact of therapy also requires exploring how cellular signaling might adapt or bypass drug inhibition. While imatinib is highly effective, prolonged exposure in oncological settings has revealed several mechanisms of drug resistance that are relevant to understanding its long‐term impact on cellular signaling. In conditions such as chronic myeloid leukemia (CML) and gastrointestinal stromal tumors (GIST), resistance typically arises from two primary pathways:

- Target Modification: Point mutations in the kinase domains (such as the *BCR‐ABL* or *KIT* genes) can reduce imatinib's binding affinity, rendering the therapy ineffective.
- Gene Amplification: Overexpression of the target gene can lead to an excess of tyrosine kinase activity that exceeds the inhibitory capacity of standard drug doses [86, 87, 88].

Beyond these target‐specific mutations, non‐genetic mechanisms of resistance have been identified which are particularly pertinent to PAH research:

1. Increased Efflux: Overexpression of ABC transporters (ABCB1 and ABCG2) can actively pump imatinib out of the cells, reducing its intracellular concentration and therapeutic efficacy.
2. Altered Metabolism: Upregulation of hepatic drug‐metabolizing enzymes may increase drug clearance, leading to subtherapeutic plasma levels [86, 87, 89, 90].

In the context of PAH, the development of resistance is still under investigation. However, understanding these pathways is crucial for the development of next‐generation tyrosine kinase inhibitors and for determining whether dose escalation—a strategy sometimes used in oncology—could safely overcome reduced sensitivity in pulmonary vascular remodeling without increasing systemic toxicity [91].

### 6.4 Pharmacodynamic Rationale: Toward Reverse Remodeling

The precise relationship between the pharmacokinetics and pharmacodynamics of imatinib in vascular tissue is still being elucidated; however, clinical experience in oncology provides a foundational understanding of its dose–response profile. In the treatment of chronic myeloid leukemia (CML) and GIST, data suggest that therapeutic efficacy is directly associated with dose intensity, with 400 mg/day established as the optimal balance between clinical response and systemic toxicity [81, 86]. While dose escalation to 600–800 mg/day has been attempted in non‐responsive oncological cases, these higher regimens often increase the incidence of adverse effects without a proportional improvement in disease control. This pharmacological threshold is highly relevant for PAH research, as it establishes the safety boundaries for treating pulmonary vascular remodeling and highlights the narrow therapeutic index that complicates systemic administration. In the context of PAH, imatinib resulted in beneficial effects by targeting the specific signaling mechanisms—primarily the PDGFRα and β receptors—that drive tissue stiffening and vessel narrowing [80]. The PDGFR system, located primarily on mesenchymal cells, transduces essential signals for cell survival, growth, and chemotaxis, facilitating an intimate epithelial‐mesenchymal cell communication that drives chronic tissue remodeling [90]. By modulating these cellular pathways, imatinib acts specifically by promoting apoptosis in hyper‐proliferative PASMCs and inhibiting the fibroblast‐to‐myofibroblast transition, thereby reducing the structural stiffness of the pulmonary arteries [90].

Unlike traditional vasodilators that target immediate vessel tone, imatinib's pharmacodynamic effect is centered on reverse remodeling, a more ambitious therapeutic goal that aims to alter the underlying disease architecture by inhibiting the proliferation and survival of pulmonary smooth muscle cells [55, 81, 92, 93, 94]. However, as observed in clinical practice, adverse events and treatment discontinuation have discouraged the long‐term pursuit of systemic therapy. This has led to the investigation of novel pharmacodynamic strategies, such as intratracheal drug administration and nanoparticle‐mediated delivery, designed to maximize local efficacy within the pulmonary vasculature while minimizing systemic exposure [80].

## 7 Evidence From Clinical Trials

Pulmonary arterial hypertension (PAH) is characterized by an increase in pulmonary vascular resistance and exists in both idiopathic and hereditary forms [12, 13, 92]. Despite the development of numerous therapies, the disease involves significant vascular remodeling, and the 5‐year mortality rate remains high, at approximately 58% [12, 13, 92, 93, 94]. Currently approved treatments primarily aim to improve symptoms and quality of life without reversing the underlying disease progression.

Imatinib, a tyrosine kinase inhibitor targeting PDGFR, c‐KIT, and BCR‐ABL, has emerged as a potential disease‐modifying agent. By arresting cell proliferation in the vascular wall and reducing vascular tone, it addresses the structural changes of PAH [52, 95, 96, 97, 98, 99]. While early studies showed improvements in the 6‐min walk distance (6MWD) and pulmonary vascular resistance (PVR), serious adverse events, such as cerebral hemorrhage, have prevented its clinical approval to date [100].

### 7.1 Precision Medicine and the PIPAH Study

Recent research has pivoted toward a precision medicine approach to mitigate toxicity. The PIPAH (Precision Imatinib for Pulmonary Arterial Hypertension) study was designed to assess the tolerability and efficacy of imatinib by identifying the maximum tolerated dose (MTD) between 100 and 400 mg/day. A key objective of PIPAH is to evaluate drug response according to individual genetic characteristics, particularly the *PDGFR*β gene variants [101, 102, 103, 104, 105, 106].

The study utilizes a two‐part design:

- Part 1: Employs a Bayesian continuous reappraisal design (CRM) to identify the MTD. This phase monitors adverse reactions closely during the first 4 weeks to allocate optimal doses for subsequent patients.
- Part 2: An open‐label efficacy study using Simon's two‐stage design to measure PVR reduction. For patients with high baseline PVR (> 1000 dyn s cm^−5^), a significant response is defined as an absolute reduction of ≥ 300 dyn s cm^−5^.

A distinguishing feature of the PIPAH study is the use of remote monitoring via CardioMEMS devices. This allows for real‐time tracking of pulmonary artery pressure and oxygen saturation, providing crucial hemodynamic data for pharmacokinetic and pharmacodynamic interpretation [96, 107, 108, 109].

### 7.2 Meta‐Analysis of Imatinib Efficacy

A significant meta‐analysis has further quantified imatinib's impact on PAH [110]. The data confirmed that imatinib effectively improves 6MWD and decreases both PVR and mean pulmonary arterial pressure (mPAP). Specifically, the reduction in PVR was substantial (DM = −396.68, 95% CI: −474.50 to −318.85).

However, the meta‐analysis highlighted a critical discrepancy: despite these hemodynamic improvements, there were no significant differences in mortality or the frequency of overall adverse reactions compared to placebo. This suggests that while imatinib is hemodynamically active, its safety profile remains a barrier to long‐term survival benefits.

### 7.3 The IMPRES Trial: Efficacy and Safety Profile

The IMPRES trial, a multicenter, randomized, double‐blind study involving 202 patients, represents the most comprehensive evaluation of imatinib in advanced PAH [111]. Participants, who were already on at least two specific therapies, received doses up to 400 mg/day. At 24 weeks, imatinib significantly improved 6MWD by a mean difference of 32 m compared to placebo (*p* = 0.002).

Beyond exercise capacity, imatinib achieved remarkable hemodynamic results:

- A reduction in PVR by approximately 32% (difference of −379 dyn s cm^−5^).
- Improvements in cardiac output (+0.88 L/min) and right atrial pressure.
- A significant decrease in NT‐proBNP levels.

Despite these gains, the trial recorded a high discontinuation rate (27%) due to adverse events such as edema, nausea, and vomiting. Most concerningly, the extension study reported subdural hematomas in 4.2% of patients, particularly those receiving concomitant anticoagulants. This safety signal underscores the need for selective monitoring and cautious administration.

### 7.4 Impact on Cardiac Function: Echocardiographic Substudy

An echocardiographic substudy within the IMPRES trial provided further insight into imatinib's effect on heart structure [111]. After 24 weeks, patients treated with imatinib showed significant improvements in right ventricular function, evidenced by increased TA S′ and a decreased RV Tei index [112–114].

Imatinib also appears to optimize left ventricular filling. The study noted increased left ventricular end‐diastolic dimensions and improved early diastolic function (*E*′), likely as a secondary effect of reduced PVR [112, 113, 114, 115, 116]. These findings suggest that imatinib may not only target the pulmonary vasculature but also alleviate the secondary cardiac strain characteristic of advanced PAH [117, 118, 119, 120, 121].

### 7.5 Long‐Term Safety and Efficacy of Imatinib

Evaluating long‐term outcomes is crucial in PAH research, as initial hemodynamic improvements may be offset by cumulative toxicity over extended treatment periods. Data regarding the long‐term profile of imatinib primarily derive from the extension phase of the IMPRES trial [55, 122, 123].

Initially, 144 of the 202 original participants entered the extension phase. While all patients eventually received imatinib (up to 400 mg/day), the results were significantly impacted by a high discontinuation rate of 31.3% due to adverse events, which limits the overall reliability of the long‐term efficacy data [123].

#### 7.5.1 Efficacy Over Time

Despite the tolerability issues, patients who remained on therapy showed sustained or increasing clinical benefits:

- 6‐min Walk Distance (6MWD): Patients treated with imatinib from the start of the core study showed an average improvement of 50 m at week 72 and 73 m at week 156, significantly exceeding the 33‐m threshold for clinical relevance [118, 123, 124].
- Functional Class: After 96 weeks, 29% of patients demonstrated an improvement in WHO functional class [123].
- Hemodynamic Stabilization: Observational data in small cohorts (*n* = 15) suggested that imatinib could stabilize or improve parameters even in patients previously refractory to triple combination therapy [119, 120].

#### 7.5.2 Critical Safety Concerns

The extension study confirmed that the risk–benefit ratio of imatinib is complicated by severe complications:

1. Subdural Hematomas (SDH): Eight cases were reported, all occurring within the first 100 days and exclusively in patients receiving concomitant anticoagulants. While regular INR monitoring may reduce this risk, the occurrence of SDH remains a distinguishing and severe adverse event of imatinib in PAH [121, 123].
2. Cardiac and Laboratory Findings: QTc prolongation was observed in several patients, though its direct correlation with mortality remains unproven. Additionally, 17 deaths occurred during the follow‐up, primarily due to the natural progression of PAH rather than direct drug toxicity [123].

#### 7.5.3 Conclusion on Long‐Term Use

In conclusion, while imatinib demonstrates potent antiproliferative activity capable of reversing vascular remodeling in animal models [95] and stabilizing advanced human PAH, its low systemic tolerability limits its suitability for routine clinical use. However, these findings have solidified the shift in PAH therapeutic strategies: moving from simple vasodilation toward targeting growth factors like PDGF to fundamentally alter the disease architecture [125, 126].

## 8 Conclusion

Originally developed as a cornerstone therapy for chronic myeloid leukemia, imatinib is now emerging as a significant proof‐of‐concept in the treatment of pulmonary arterial hypertension (PAH). Its primary value lies in its ability to target the cellular mechanisms of vascular remodeling, particularly in patients who remain refractory to conventional vasodilator therapies.

Clinical evidence, most notably from the IMPRES trial, has demonstrated that imatinib can significantly lower pulmonary vascular resistance and improve exercise capacity (6MWD). However, its clinical application remains hindered by a complex safety profile, specifically the risk of systemic toxicity and intracranial hemorrhage, which have precluded its approval as a first‐line treatment.

The future of imatinib in PAH management appears to be heading in two complementary directions:

- Precision Medicine: Pharmacogenetic studies, such as the PIPAH trial, are essential to identifying “responders” based on individual genetic profiles (e.g., *PDGFR*β variants), potentially allowing for a more targeted and safer use of the drug.
- Novel Delivery Systems: The exploration of inhaled imatinib represents a promising strategy to deliver the drug directly to the pulmonary vasculature, maximizing local anti‐proliferative effects while minimizing systemic exposure and associated adverse events.

In conclusion, while imatinib is not yet a definitive cure, it represents a fundamental shift in PAH research—moving beyond symptom management toward the goal of reversing the underlying structural pathology. Its journey highlights both the potential of drug repurposing and the ongoing challenges of balancing potent efficacy with patient safety in a seriously debilitating disease.

## Disclosure

Generative AI tools were used only for grammar and language corrections. All content, ideas, and analyses are solely the work of the authors.

## Conflicts of Interest

The authors declare no conflicts of interest.

## Data Availability

Data sharing not applicable to this article as no datasets were generated or analysed during the current study.
